# Recent estimates and predictions of 5-year survival rate in patients with pancreatic cancer: A model-based period analysis

**DOI:** 10.3389/fmed.2022.1049136

**Published:** 2022-12-08

**Authors:** Jing Li, Yunmei Li, Chong Chen, Jiayu Guo, Mengmeng Qiao, Jun Lyu

**Affiliations:** ^1^Department of Clinical Research, The First Affiliated Hospital of Jinan University, Guangzhou, Guangdong, China; ^2^School of Public Health, Shaanxi University of Chinese Medicine, Xianyang, Shaanxi, China; ^3^School of Basic Medicine and Public Health, Jinan University, Guangzhou, Guangdong, China; ^4^Guangdong Provincial Key Laboratory of Traditional Chinese Medicine Informatization, Guangzhou, Guangdong, China

**Keywords:** pancreatic cancer, period analysis, relative survival rate, prediction, SEER

## Abstract

**Background:**

The 5-year survival rate for pancreatic cancer (PC) is incredibly low, resulting in this often being a fatal disease. Timely and accurate assessment of the survival rate and prognosis of patients with PC is of great significance for the development of new programs for prevention, monitoring, and treatment.

**Methods:**

Period analysis and further stratified analysis were used to determine the 5-year relative survival rate (RSR) of patients with PC from 2002 to 2016 using the Surveillance, Epidemiology, and End Results (SEER) project database of the National Cancer Institute. Based on this, a generalized linear model was created to predict the survival rate of patients from 2017 to 2021.

**Result:**

During 2002–2016, the 5-year RSR of patients with PC increased from 7.9 to 23.7%. The generalized linear model predicted that the survival rate had increased to 33.9% during 2017–2021, and hence, it was still unacceptably low. The survival rate of patients aged ≥75 years at diagnosis was the lowest among all age groups and was predicted to be only 21.4% during 2017–2021. Notably, the survival rate of patients with differentiation grade III at diagnosis remains particularly low at 7.6%.

**Conclusion:**

The survival rates of patients with PC, although slightly improved, remain extremely low. Timely assessment of the trend of survival rate changes in patients with PC further improves the prognosis of tumor patients and provides data support for relevant medical works to formulate effective tumor prevention and control policies.

## Introduction

Pancreatic cancer (PC) has a poor prognosis and remains one of the most deadly types of cancer. The global cancer statistics for 2020 indicate that PC deaths (466,003) were almost equal to the number of cases (496,773) (1), making it the seventh most common cause of cancer death worldwide (2). PC is predicted to soon overtake breast cancer as the third most common cause of cancer-related deaths (3).

Only 5% of PC cases are diagnosed early, and thus most patients are in the locally advanced or metastatic stage (4). More than 90% of people with PC die from the disease due to its characteristics, which include its rapid spread and difficulty in treatment (5). Further analyses of the factors influencing PC are, therefore, crucial for clinicians and patients to improve its prevention, timely diagnosis, treatment, and survival. However, since the early stages of the disease are asymptomatic, most patients receive a diagnosis when it is already in the advanced stage, and there are currently no recognized early screening techniques. Primary prevention through clinical research, changing controllable risk factors, and optimizing medical policies may, therefore, be the most effective way to reduce the burden of PC (6).

Relative survival is often replaced by absolute survival in population-based cancer surveillance. The relative survival rate (RSR) is an important indicator for assessing the treatment effect and clinical prognosis of patients with cancer (7). Relative survival refers to the ratio of actual survival to the expected survival in the absence of cancer. Expected survival rates were calculated based on the current life table statistics of the underlying population adjusted for sex, age, and calendar years. Relative survival estimation was performed using the Ederer II and Hakulinen methods (8). Although studies have evaluated the worldwide and regional epidemiological characteristics of PC (3), few studies have evaluated the relationship between factors and disease prognosis and calculated future survival. Compared with traditional cohorts and complete analyses, the predicted future survival rate based on the period analysis method in the generalized linear model was closer to the actual observed survival rate (9). We used period analysis for the first time in PC.

To provide oncologists with a scientific foundation for tumor prevention and treatment strategies, this study performed period analyses using data from the Surveillance, Epidemiology, and End Results (SEER) database to predict the survival rate of patients with PC.

## Materials and methods

### Data source

We extracted information from the records of patients with PC in the SEER project database of the National Cancer Institute. The SEER database is the definitive source for cancer statistics (10) and regularly collects information such as patient’s demographic data and primary tumor locations (11–13).

The inclusion criteria were (1) diagnosis between January 1997 and December 2016 and (2) morphological codes 8140/3–8384/3 and anatomical codes C25.0–C25.9 of the third revision International Classification of Diseases for Oncology. The exclusion criteria were (1) unknown variables or incomplete follow-up information, (2) lost cases, and (3) or incomplete pathological data or diagnostic certificates. We ultimately extracted 39,700 patients with PC who met the inclusion and exclusion criteria.

### Data sorting

The variables extracted in this study included sex, race, age at diagnosis, socioeconomic status, differentiation grade, SEER stage, radiation therapy, chemotherapy, and year and month of diagnosis. The race was divided into three groups, namely, White, Black, and Others (American Indian/Alaska Native and Asian/Pacific Islander). Age at diagnosis was divided into five groups, namely, 0–44, 45–54, 55–64, 65–74, and ≥75 years according to the International Cancer Survival Standards (ICSS) for age-standardized survival. Socioeconomic status was divided into four groups, namely, high income (<7%), upper-middle income (≥7 and <9.62%), lower-middle income (≥9.62 and <13.15%), and low income (≥13.15%), based on the poverty rate in the area of residency (14). According to the degree of tumor differentiation at the time of diagnosis, patients were classified into four grades, namely, GI (highly differentiated), GII (moderately differentiated), GIII (poorly differentiated), and GIV (undifferentiated). Four SEER stages were used, namely, local, regional, and distant metastases, and unknown. Clinical treatment options included whether to accept the use of radiation therapy and chemotherapy.

### Statistical analysis

This article adopted the method of period analysis proposed by Brenner and Gefeller in 1996 to evaluate and analyze the RSR and trend of patients with PC diagnosed during three time periods, namely, 2002–2006, 2007–2011, and 2012–2016 (15). A generalized linear model was constructed based on the period analysis to predict the future survival rate of patients with PC diagnosed during 2017–2021. Relative survival is the ratio of the observed survival to the expected survival and is expressed as R_*i*_ = S_*k*_/S_k_* where S_*k*_ and S_k_* represent the true and expected survival rates, respectively, and k = 5 was used to calculate the 5-year RSR (8, 9, 16, 17). We used the log-rank test with a significance threshold of 0.05 to evaluate the difference between these curves. The Cox regression analysis was used to determine whether age, sex, race, socioeconomic status, differentiation grade, SEER stage, radiation therapy, and chemotherapy were independent risk factors. The above mentioned evaluation and data analysis were completed using the periodR, survival, survminer, IPWsurvival, ggprism, ggplot2, reshape2, ggalt, cowplot, plyr, and foreign packages of the R software.

## Results

Among the 39,700 patients included in the study, 6,074, 9,777, 11,162, and 12,687 cases were registered in the four observation periods of 1997–2001, 2002–2006, 2007–2011, and 2012–2016 accounting for 15.30, 24.63, 28.12, and 31.96% of the population, respectively. There were more male patients than female patients. The median age at diagnosis was 68 years. Among the age quartiles, the 25th and 75th percentiles represented the ages of 59 and 76 years, respectively. Regarding socioeconomic status, low-income patients comprised the most cases in each observation period. Among the differentiation grades, cases were mostly concentrated in PC grades GII and GIII, namely, moderately differentiated and poorly differentiated. Regarding the SEER stage, patients with regional and distant metastases at the time of diagnosis were the most common, accounting for 83.47%. Only 0.81% of patients refused radiation therapy. The ratio between the number of patients who accepted chemotherapy use and the number of patients who refused or had an unknown decision was 0.96:1. These data are listed in Table 1.

**TABLE 1:** Distribution of pancreatic cancer (PC) cases from 1997 to 2016.

Variables	1997–2001	2002–2006	2007–2011	2012–2016	*P*-value
	*n* (%)	*n* (%)	*n* (%)	*n* (%)	
All	6,074	9,777	11,162	12,687	
Sex					0.009
Male	3,113 (51.3)	4,969 (50.8)	5,715 (51.2)	6,709 (52.9)	
Female	2,961 (48.7)	4,808 (49.2)	5,447 (48.8)	5,978 (47.1)	
Race					<0.001
White	4,966 (81.8)	7,874 (80.5)	8,909 (79.8)	10,146 (80.0)	
Black	684 (11.3)	1,260 (12.9)	1,408 (12.6)	1,488 (11.7)	
Other races	424 (7.0)	643 (6.6)	845 (7.6)	1,053 (8.3)	
Diagnosis age					<0.001
≤44	223 (3.7)	384 (3.9)	444 (4.0)	581 (4.6)	
45–54	701 (11.5)	1,196 (12.2)	1,381 (12.4)	1,441 (11.4)	
55–64	1,294 (21.3)	2,285 (23.4)	2,850 (25.5)	3,383 (26.7)	
65–74	1,972 (32.5)	2,953 (30.2)	3,236 (29.0)	4,026 (31.7)	
≥75	1,884 (31.0)	2,959 (30.3)	3,251 (29.1)	3,256 (25.7)	
Socioeconomic status					<0.001
High income	1,621 (26.7)	2,428 (24.8)	2,789 (25.0)	3,078 (24.3)	
Upper-middle income	1,529 (25.2)	2,374 (24.3)	2,738 (24.5)	3,264 (25.7)	
Lower-middle income	849 (14.0)	1,632 (16.7)	1,872 (16.8)	2,237 (17.6)	
Low income	2,075 (34.2)	3,343 (34.2)	3,763 (33.7)	4,108 (32.4)	
Differentiation grade					<0.001
GI	791 (13.0)	1,331 (13.6)	1,995 (17.9)	3,324 (26.2)	
GII	2,392 (39.4)	4,009 (41.0)	4,422 (39.6)	4,748 (37.4)	
GIII	2,756 (45.4)	4,214 (43.1)	4,555 (40.8)	4,373 (34.5)	
GIV	135 (2.2)	223 (2.3)	190 (1.7)	242 (1.9)	
SEER stage					<0.001
Localized	498 (8.2)	930 (9.5)	1,260 (11.3)	2,573 (20.3)	
Regional	2,278 (37.5)	4,094 (41.9)	4,961 (44.4)	5,165 (40.7)	
Distant	2,911 (47.9)	4,343 (44.4)	4,675 (41.9)	4,712 (37.1)	
Unknown	387 (6.4)	410 (4.2)	266 (2.4)	237 (1.9)	
Radiation therapy					<0.001
Yes	1,481 (24.4)	2,412 (24.7)	2,423 (21.7)	1,833 (14.4)	
Refused	60 (1.0)	85 (0.9)	97 (0.9)	78 (0.6)	
Unknown	4,533 (74.6)	7,280 (74.5)	8,642 (77.4)	10,776 (84.9)	
Chemotherapy					<0.001
Yes	2,697 (44.4)	4,644 (47.5)	5,909 (52.9)	6,230 (49.1)	
No/Unknown	3,377 (55.6)	5,133 (52.5)	5,253 (47.1)	6,457 (50.9)	

The patient population distribution of the variables in each observation period is not 100% due to rounding.

Meanwhile, we performed univariate and multifactorial Cox regression analyses on survival data of patients diagnosed with PC during 2002–2016, which were used to explore the relationship between each factor and disease. The results indicated that all variables were independent risk factors in the univariate analysis (*P* < 0.05 for all variables, Table 2). In the multivariate analysis, two variables, other races and refused radiation therapy, were not statistically significant (*P* > 0.05, Table 2).

**TABLE 2:** Summary of Cox regression analysis of survival time of patients with pancreatic cancer (PC).

Variables	Univariate			Multivariate		
		
	95% CI	HR	*P*	95% CI	HR	*P*
Diagnosis age	1.026–1.028	1.027	<0.001	1.024–1.026	1.025	<0.001
Sex						
Male		1.0			1.0	
Female	0.957–0.999	0.978	0.040	0.927–0.969	0.948	<0.001
Race						
White		1.0			1.0	
Black	1.104–1.180	1.142	<0.001	1.158–1.239	1.197	<0.001
Other races	0.886–0.965	0.924	<0.001	0.932–1.016	0.973	0.209
Socioeconomic status						
High income		1.0			1.0	
Upper-middle income	1.028–1.093	1.060	<0.001	1.003–1.067	1.034	0.033
Lower-middle income	1.092–1.170	1.131	<0.001	1.102–1.181	1.141	<0.001
Low income	1.198–1.269	1.233	<0.001	1.120–1.188	1.154	<0.001
Differentiation grade						
GI		1.0			1.0	
GII	2.120–2.275	2.196	<0.001	2.025–2.178	2.100	<0.001
GIII	3.249–3.486	3.365	<0.001	2.937–3.162	3.047	<0.001
GIV	2.928–3.437	3.172	<0.001	2.489–2.926	2.699	<0.001
SEER stage						
Localized		1.0			1.0	
Regional	1.932–2.102	2.015	<0.001	1.671–1.822	1.745	<0.001
Distant	4.229–4.599	4.410	<0.001	3.803–4.149	3.972	<0.001
Unknown	3.445–3.952	3.690	<0.001	2.526–2.901	2.707	<0.001
Radiation therapy						
Yes		1.0			1.0	
Refused	1.490–1.881	1.674	<0.001	0.991–1.255	1.115	0.071
Unknown	1.310–1.381	1.345	<0.001	1.005–1.066	1.035	0.022
Chemotherapy						
Yes		1.0			1.0	
No/Unknown	1.185–1.238	1.211	<0.001	1.545–1.623	1.584	<0.001

95% CI, 95% confidence interval; HR, hazard risk.

**FIGURE 1:**
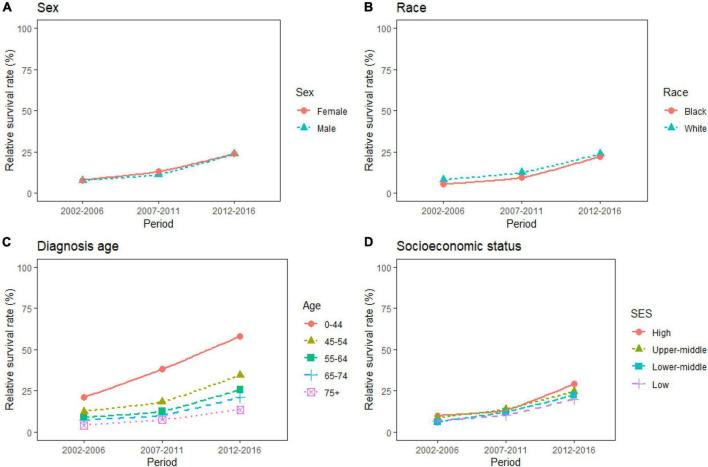
**(A–D)** Five-year relative survival rates according to sex (female and male), race (black and white), diagnosis age (0–44, 45–54, 55–64, 65–74, and 75+), and socioeconomic status (high income, upper-middle income, lower-middle income, and low income) for patients with pancreatic cancer (PC) from 2002 to 2016.

Over the 15-year period from 2002 to 2016, the overall RSR for patients with PC ranged from 7.9 to 23.7%. The generalized linear model predicted an overall RSR of 33.9% in patients with PC during 2017–2021. The model predicted survival rates of 34.0 and 34.2% for male and female patients with PC during 2017–2021, respectively. During 2002–2016, the survival rates were higher in White patients than in Black patients. The survival rates for White and Black patients during 2017–2021 were predicted to be 35.5 and 34.0%, respectively. These data are listed in Table 3, and the trends are shown in Figures 1A,B.

**TABLE 3:** The survival rate of patients with pancreatic cancer (PC) by all variables from 2002 to 2016 and the survival rate prediction of different variables from 2017 to 2021.

Variables	2002–2006		2007–2011		2012–2016		2017–2021
				
	RSR (%)	SE	RSR (%)	SE	RSR (%)	SE	RSR (%)
All	7.9	0.3	12.1	0.4	23.7	0.5	33.9
Sex							
Male	7.8	0.5	11.3	0.5	23.7	0.6	34.0
Female	8.0	0.5	13.0	0.5	23.8	0.7	34.2
Race							
White	8.2	0.4	12.5	0.4	23.8	0.5	35.5
Black	5.4	0.8	9.4	0.9	22.2	1.3	34.0
Other races	8.9	1.3	12.8	1.5	25.4	1.7	33.5
Diagnosis age							
≤44	21.0	2.3	38.0	2.6	58.2	2.4	92.1
45–54	12.5	1.1	18.3	1.2	34.5	1.4	48.9
55–64	8.8	0.7	12.5	0.7	25.7	0.9	38.1
65–74	7.2	0.6	10.1	0.6	20.9	0.8	27.8
≥75	4.0	0.5	7.4	0.6	13.5	0.8	21.4
Socioeconomic status							
High income	9.9	0.7	13.2	0.8	29.1	1.0	40.4
Upper-middle income	8.7	0.7	13.8	0.8	24.5	0.9	34.4
Lower-middle income	6.1	0.8	12.1	0.9	22.3	1.1	37.3
Low income	6.8	0.5	10.2	0.6	19.9	0.7	28.0
Differentiation grade							
GI	18.2	1.3	37.0	1.4	60.8	1.1	93.1
GII	9.0	0.6	10.7	0.5	17.6	0.7	20.7
GIII	3.8	0.4	4.6	0.4	7.1	0.4	7.6
GIV	5.4	1.8	7.5	2.2	8.4	2.3	7.7
SEER stage							
Localized	22.9	1.7	37.5	1.7	70.6	1.3	96.2
Regional	10.4	0.6	13.5	0.6	21	0.6	26.8
Distant	2.7	0.3	4.9	0.4	7.7	0.4	10.5
Unknown	5.1	1.1	7.4	1.7	12.1	2.5	17.1
Radiation therapy							
Yes	11.0	0.7	11.2	0.7	17.2	0.9	19.2
Refused	3.5	2.4	6.6	4.0	7.6	2.8	7.8
Unknown	7.0	0.4	12.9	0.4	26.1	0.5	40.1
Chemotherapy							
Yes	7.9	0.5	9.2	0.4	12.7	0.5	14.3
No/Unknown	7.8	0.4	16.0	0.6	38.1	0.7	67.2

RSR, relative survival rate; SE, standard error.

**FIGURE 2:**
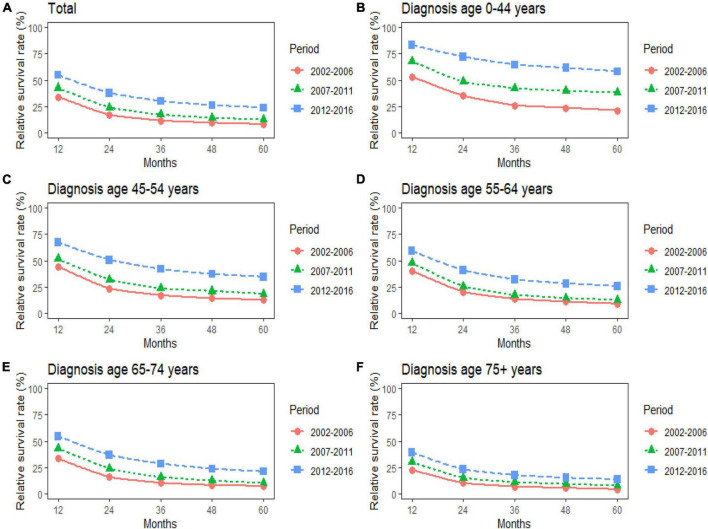
**(A–F)** Trends in 5-year relative survival rates for patients with pancreatic cancer (PC) from 2002 to 2016. Data are shown by age group (total and age 0–44, 45–54, 55–64, 65–74, and 75+ years) and calendar period.

**FIGURE 3:**
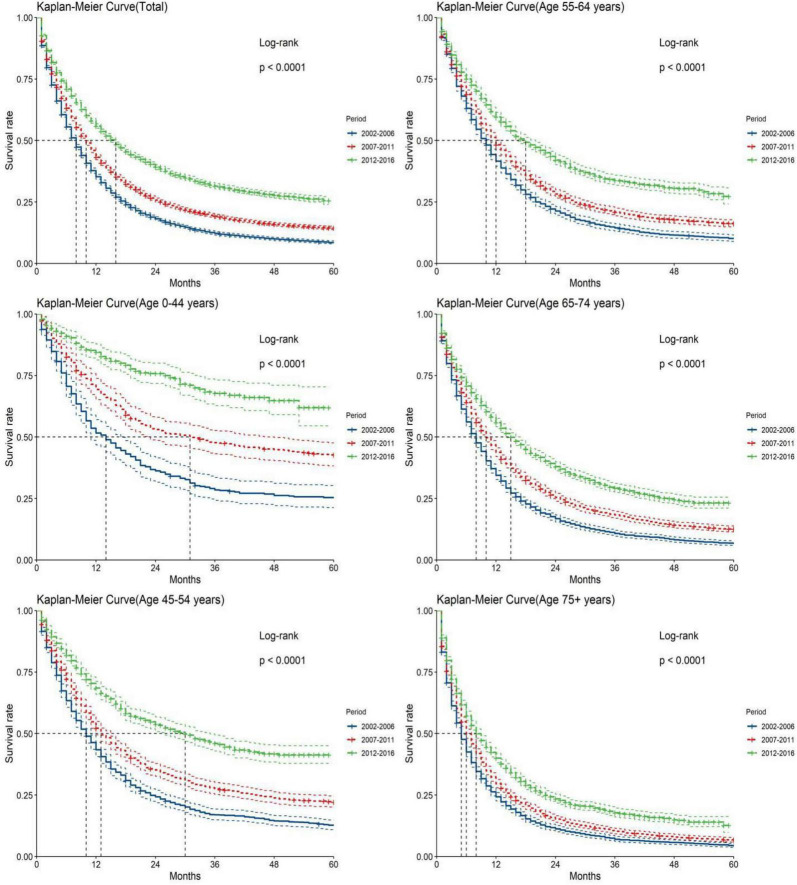
Kaplan-Meier survival analyses for patients with pancreatic cancer (PC) from 2002 to 2016. Data are shown by age group (total and age 0–44, 45–54, 55–64, 65–74, and 75+ years) and calendar period.

**FIGURE 4:**
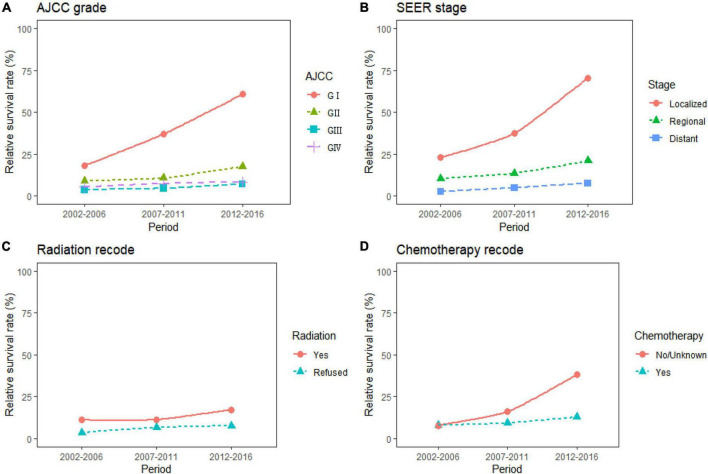
Five-year relative survival rates according to differentiation grade (**A**, GI, GII, GIII, and GIV), SEER stage (**B**, localized, regional, distant), radiation therapy (**C**, yes, refused), and chemotherapy (**D**, yes, no/unknown) for patients with pancreatic cancer (PC) from 2002 to 2016.

The study predicted that the survival rate of patients with PC aged ≥75 years at diagnosis during 2017–2021 would be only 21.4%. There was an inverse relationship between age at diagnosis and median survival time. At the same time, RSR in all age groups showed an increasing trend over time during the 15-year observation period from 2002 to 2016. The survival rate of PC decreased significantly with age. From 2012 to 2016, the 5-year RSR for patients with PC in the age group 0–44 years at diagnosis was 58.2%, compared with 13.5% for patients in the age group ≥75 years at diagnosis (Figure 2). The same trend in patient survival was found in all other years and age groups. Kaplan–Meier survival analysis indicated an increasing trend in median survival time over time for PC in each age group of diagnosis during the period 2002–2016. In addition, the median survival time of patients decreased significantly with age (Figures 2, 3). Survival rates for both male and female patients in all diagnostic age groups showed a significant upward trend over time (Supplementary Table 1 and Supplementary Figure 1A). Patients with higher socioeconomic statuses had higher survival rates. We predicted that the survival rates of high- and low-income patients with PC would be 40.4 and 28.0% during 2017–2021, respectively, giving a difference of 12.4%. The changing trends are shown in Figures 1C,D. Survival rates for Whites and Blacks of all economic statuses showed an increasing trend over time (Supplementary Table 2 and Supplementary Figures 1B, 2). The survival rate of patients with PC improved for all differentiation grades between 2002 and 2016. The survival rates of patients in grades GI and GIII during 2017–2021 were predicted to be 93.1 and 7.6%, respectively. The study predicted that the survival rates of patients with local and distant metastases at diagnosis would be 96.2 and 10.5% during 2017–2021, respectively. Survival of patients with local, regional, and distant metastases at all different grades of differentiation showed an increasing trend over time (Supplementary Table 3 and Supplementary Figure 1C). The changing trends are shown in Figures 4A,B.

During the 15-year observation period from 2002 to 2016, the survival rates were higher in patients with PC who received radiation therapy than in those who did not. The study predicted that the survival rates of patients who received and refused radiation therapy would be 19.2 and 7.8% during 2017–2021, respectively. The survival rate of patients who received chemotherapy was predicted to be 14.3%. Survival rates for patients treated with radiation therapy and chemotherapy showed an increasing trend over time (Supplementary Table 4 and Supplementary Figure 1D). The trends are shown in Figures 4C,D.

## Discussion

Pancreatic cancer is an increasingly common cancer worldwide, often presenting at an advanced stage and as an adenocarcinoma (85%) (18). PC occurrence rates continue to increase, but survival rates have barely changed (3). It is, therefore, very important to timely analyze and predict the survival rate of patients with PC.

The number of confirmed PC cases has increased each year, which results in serious family and economic burdens. The global burden of PC has more than doubled over the past 25 years (19). This might be related to risk factors such as an aging global population, smoking, obesity, diabetes, high-fat diets, and alcohol consumption (20–23). Although the trend is increasing, it is still relatively low, which indicates that there are still many patients with PC who do not survive for more than 5 years. During 2002–2016, the ratio of male to female patients was approximately 1.07:1, and the survival rate of male patients was consistently lower than that of female patients. This could be a result of differences in lifestyles and behaviors between the two sexes. Men are more likely to consume excess amounts of high-calorie foods and red meat than women. They are also more likely to smoke, drink alcohol, and engage in other unhealthy behaviors than women (18, 24). While alcohol makes PC more sensitive to other risk factors, smoking is a known risk factor for the disease (25). These two factors work together to increase the probability of developing a PC. Smoking and drinking habits often co-exist, with smoking rates increasing with alcohol consumption (26). It is noteworthy that there is a lack of a relationship between reproductive variables and PC in women (27). This indicates that environmental factors may cause the difference in occurrence between men and women.

The risk of death from PC increases sharply with age. According to this study, the survival rate of people with PC older than 75 years was predicted to be only 21.4% during 2017–2021, which was 70.7% lower than the survival rate of their counterparts younger than 44 years. The aging of the population is the main reason for the current low survival rate (28). The deterioration of autoimmunity in the elderly and the complexity of comorbidities reduce the probability of survival. The race is a recognized risk factor for PC. Lower survival rates for Black patients are a result of the increased propensity for PC being diagnosed at an advanced stage and the resulting lower feasibility of surgical treatment. This may be attributed to differences in diet, alcohol, smoking, and other factors between Black patients and those of other races, and particularly to genetic differences in specific races. Due to these variations, there is a higher probability of developing mutations from known poisons, like the capacity to detoxify tobacco products. A study that compared tumor gene mutations and biomarker immune expression between races found that Black patients had significantly higher KRAS mutation rates for valine and lower KRAS mutation rates for cysteine; Fas expression was also lower in Black patients, while the immune expression of HER2 tended to be elevated (29, 30). These factors explain the lower survival rate for Black patients. More economically disadvantaged regions have more PC cases and a higher death rate, which might be due to lifestyle habits, differences in the management of cancer surveillance, and registration systems in their areas of residence (31, 32).

This study found that the highest proportion of patients was in GII and GIII at the time of diagnosis. The RSRs for patients with PC in GI and GIV at diagnosis during 2017–2021 was predicted to be 93.1 and 7.7%, respectively. Tumors grow and spread more rapidly in patients already in a moderately or poorly differentiated grade at diagnosis, and 80–90% of these patients have unresectable tumors, which results in lower survival rates (18, 33, 34). This study found that more than 50% of patients had already progressed to regional or distant metastases at the time of diagnosis, with only a small proportion having local metastases. It was also demonstrated that even when patients were in the local metastasis stage at the time of diagnosis and could undergo local tumor surgical resection with a microscopically negative margin (R0), the recurrence rate was still high. The recurrence rate was 62% in a study of 175 individuals who underwent R0 resection (29, 35). Patients with PC have non-specific reactions to the disease and the tumor tends to invade major blood vessels, making it inoperable and reducing the survival probability (36). Primary prevention should, therefore, be performed to improve the probability of survival through the early screening and detection of people with a high risk of PC through surveillance.

The stage of disease progression determines the optimal treatment option. Over the 15-year period from 2002 to 2016, patients with PC who received radiation therapy consistently had higher 5-year survival rates than those who refused this treatment. The predicted survival results for 2017–2021 also maintained that trend. Radiation therapy uses X-rays to destroy cancer cells so that they cannot proliferate and grow. It is important to note that patients who undergo radiation therapy should also receive multiple courses of post-treatment care to repair the surrounding tissues that have also been severely damaged (37). Patients with PC who received chemotherapy had lower survival rates than those who refused it. This may be related to chemotherapy causing fibrosis within the pancreas, resulting in complications that delay or prevent the progression of other treatments for these patients (30, 38).

Our study provides a timely update of survival estimates for patients with PC and a comprehensive overview of the survival and prognosis of patients with PC from 2002 to 2016. This study shows that both differentiation grade and stage at diagnosis of patients with PC are important factors affecting disease prognosis. White patients had higher survival rates than Black patients, and these findings are consistent with previous studies (39). Through research, we can improve our understanding of people at high risk for PC and modifiable and non-modifiable risk factors. As a result, clinicians and public health agencies can make more informed decisions about patients’ risk, prevention, and monitoring needs (3).

## Conclusion

The 5-year RSR of patients with PC grew marginally from 7.9 to 23.7% during the 15-year period from 2002 to 2016. The RSR for patients during 2017–2021 was predicted to be 33.9%. The RSRs for patients with PC remain low. Predictions for trends and results of survival rates indicated a gradual increase in the differences in survival rates with the age at diagnosis. Notably, the survival rate of patients in GIII and patients with distant metastases at the time of diagnosis was very low. Differences remain between patients who received and did not receive radiation treatment. The change in survival rates over the past 15 years was analyzed, and the future survival rates for patients with PC during 2017–2021 were predicted in order to provide theoretical support for improving the clinical management and treatment options for these patients.

### Limitations

This study had some limitations: (1) it only included patients with complete information, resulting in the exclusion of patients with some missing information, which may have introduced selection bias; (2) the SEER database is derived from the US population and so lacks data from other regions; and (3) retrospective data were analyzed, so the study was subject to retrospective bias.

## Data availability statement

The original contributions presented in this study are included in the article/Supplementary material, further inquiries can be directed to the corresponding author.

## Ethics statement

All procedures performed in this study were in accordance with the principles outlined in the 1964 Declaration of Helsinki and its later amendments. Institutional review board approval and informed consent were not required in this study because SEER research data are publicly available and all patient data are de-identified.

## Author contributions

JiL and YL created the study protocol, performed the statistical analyses, and wrote the first manuscript draft. CC conceived the study and critically revised the manuscript. JG and MQ assisted with the study design and performed data collection. JiL assisted with data collection and manuscript editing. JuL contributed to data interpretation and manuscript revision. All authors read and approved the final manuscript.
