# Supplementary material for: Recent estimates and predictions of 5-year survival rate in patients with pancreatic cancer: A model-based period analysis
Source: Front Med (Lausanne). 2022 Dec 8;9:1049136. doi: 10.3389/fmed.2022.1049136 (PMC9773388; doi:10.3389/fmed.2022.1049136)
Supplement: Supplementary file 1 [file Data_Sheet_1.pdf]

Supplementary Table 1. Five-year relative survival rate of patients with pancreatic cancer according to sex, age group, and calendar period from 2002 to 2016.

Observation period	Diagnosis age	Male		Female	
		RSR (%)	SE	RSR(%)	SE
**2002-2006**					
	≤44	17.0	2.8	26.1	3.7
	45-54	11.9	1.5	13.2	1.7
	55-64	7.5	0.9	10.6	1.2
	65-74	7.5	0.8	6.9	0.8
	≥75	4.3	0.8	3.8	0.6
**2007-2011**					
	≤44	37.4	3.8	38.4	3.6
	45-54	15.6	1.5	21.9	1.9
	55-64	10.6	0.9	14.7	1.1
	65-74	9.8	0.9	10.5	0.9
	≥75	7.0	0.9	7.7	0.8
**2012-2016**					
	≤44	55.3	3.5	60.8	3.3
	45-54	29.7	1.8	40.6	2.1
	55-64	24.8	1.2	26.9	1.3
	65-74	22.2	1.1	19.4	1.1
	≥75	15.0	1.3	12.4	1.0

Note: RSR, relative survival rate; SE, standard error.

Supplementary Table 2. Five-year relative survival rate of patients with pancreatic cancer according to race, socioeconomic status, and calendar period from 2002 to 2016.

Observation period	SES	White		Black		Other races	
		RSR(%)	SE	RSR(%)	SE	RSR(%)	SE
**2002-2006**							
	High income	10.4	0.8	7.6	2.8	6.6	1.8
	Upper-middle income	8.6	0.7	8.0	2.8	12.4	3.6
	Lower-middle income	6.1	0.8	4.4	1.7	15.9	5.5
	Low income	7.1	0.6	4.8	1.0	8.7	2.4
**2007-2011**							
	High income	13.1	0.8	12.6	3.3	15.2	2.5
	Upper-middle income	14.0	0.8	10.4	2.4	15.3	3.4
	Lower-middle income	12.5	1.0	12.2	2.6	6.2	3.1
	Low income	10.8	0.7	7.5	1.1	11.1	2.5
**2012-2016**							
	High income	28.7	1.1	28.8	4.2	31.3	2.7
	Upper-middle income	24.6	1.0	27.9	3.7	18.3	3.5
	Lower-middle income	22.1	1.2	21.7	2.4	25.2	5.0
	Low income	19.9	0.9	18.8	1.7	21.5	3.2

Note: RSR, relative survival rate; SE, standard error; SES, socioeconomic status.

Supplementary Table 3. Five-year relative survival rate of patients with pancreatic cancer according to Differentiation grade, SEER stage, and calendar period from 2002 to 2016.

Observation period	grade	Localized		Regional		Distant	
		RSR(%)	SE	RSR(%)	SE	RSR(%)	SE
**2002-2006**							
	G I	44.4	4.2	15.1	1.8	11.6	1.8
	G II	19.8	2.5	12.0	0.9	2.8	0.5
	G III	12.3	2.4	6.6	0.8	0.9	0.2
	GIV	0	0	14.8	5.4	2.2	1.6
**2007-2011**							
	G I	68.8	3.0	30.5	2.1	23.0	2.1
	G II	26.3	2.4	12.8	0.8	4.2	0.6
	G III	12.0	2.2	7.9	0.7	1.1	0.3
	GIV	13.8	9.2	14.2	4.8	1.9	1.5
**2012-2016**							
	G I	88.5	1.4	48.9	1.9	25.9	2.0
	G II	55.5	2.8	18.3	0.9	7.5	0.8
	G III	18.0	2.5	11.0	0.8	2.5	0.4
	GIV	21.4	11,7	10.7	4.3	6.3	2.9

Note: RSR, relative survival rate; SE, standard error.

Supplementary Table 4. Five-year relative survival rate of patients with pancreatic cancer according to radiation therapy, chemotherapy, and calendar period from 2002 to 2016.

Observation period	Radiation	Chemotherapy	
		Yes	No/Unknown
**2002-2006**	Yes	11.5±0.8	7.5±1.6
	Refused	0±0	3.4±2.3
**2007-2011**	Yes	11.4±0.7	9.3±2.0
	Refused	8.9±11.1	5.7±4.0
**2012-2016**	Yes	17.6±1.0	14.1±2.8
	Refused	13.1±7.2	5.1±2.7

Note: RSR, relative survival rate; SE, standard error; Data are means ± standard error of the mean.

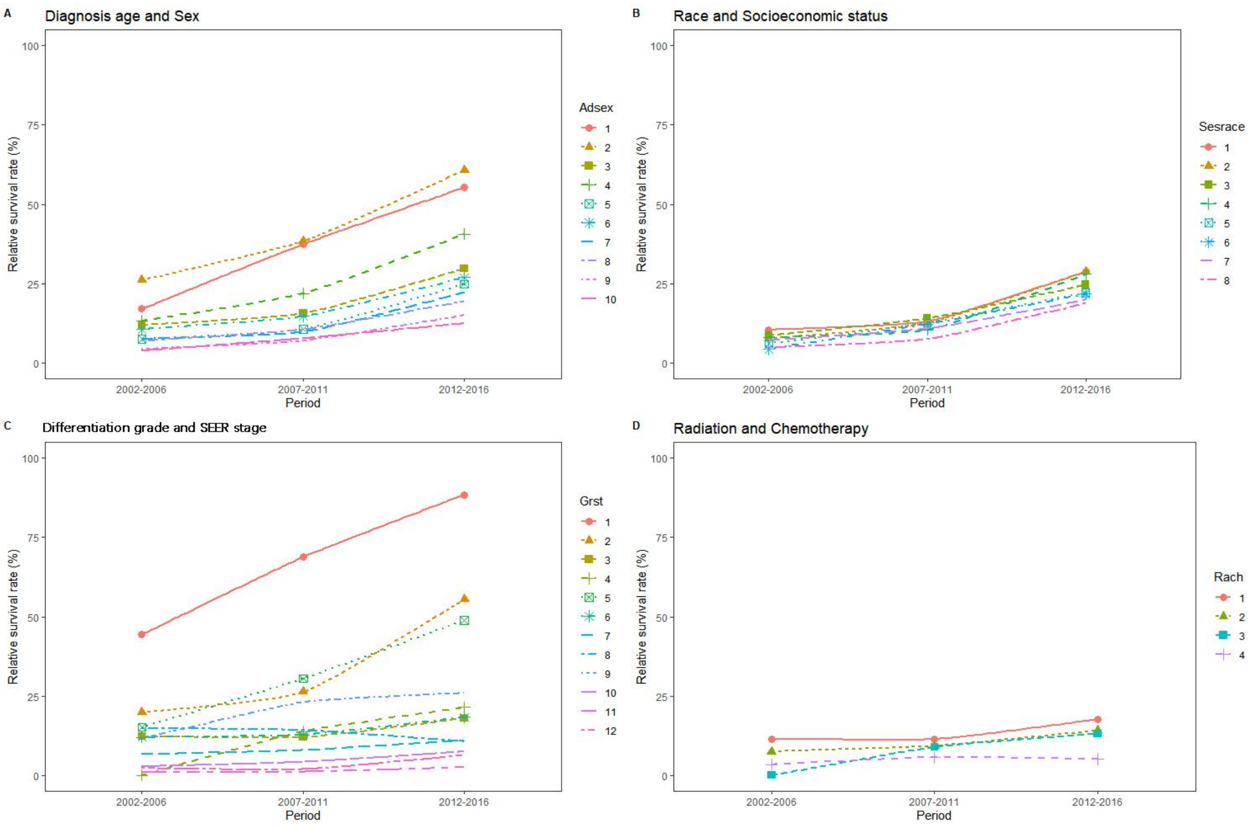

Supplementary Figure 1. Trends in 5-year relative survival rates according to all variables for patients with pancreatic cancer (PC) from 2002 to 2016.

Notes:

A:

1: 0-44, Male; 2: 0-44, Female; 3: 45-54, Male; 4: 45-54, Female; 5: 55-64, Male; 6: 55-64, Female; 7: 65-74, Male; 8: 65-74, Female; 9: 75+, Male; 10: 75+,Female.

B:

1: High, White; 2: High, Black; 3: Upper-middle, White; 4: Upper-middle, Black; 5: Lower-middle, White; 6: Lower-middle, Black; 7: Low, White; 8: Low, Black.

C:

1: GⅠ, Localized; 2: GⅡ, Localized; 3: GⅢ, Localized; 4: GⅣ, Localized; 5: GⅠ, Regional; 6: GⅡ, Regional; 7: GⅢ, Regional; 8: GⅣ, Regional; 9: GⅠ, Distant; 10: GⅡ, Distant; 11: GⅢ, Distant; 12: GⅣ, Distant.

D:

1: Radiation therapy, Chemotherapy; 2: Radiation therapy, Refused chemotherapy or Unknown; 3: Refused radiation therapy, Chemotherapy; 4: Refused radiation therapy, Refused chemotherapy or Unknown.

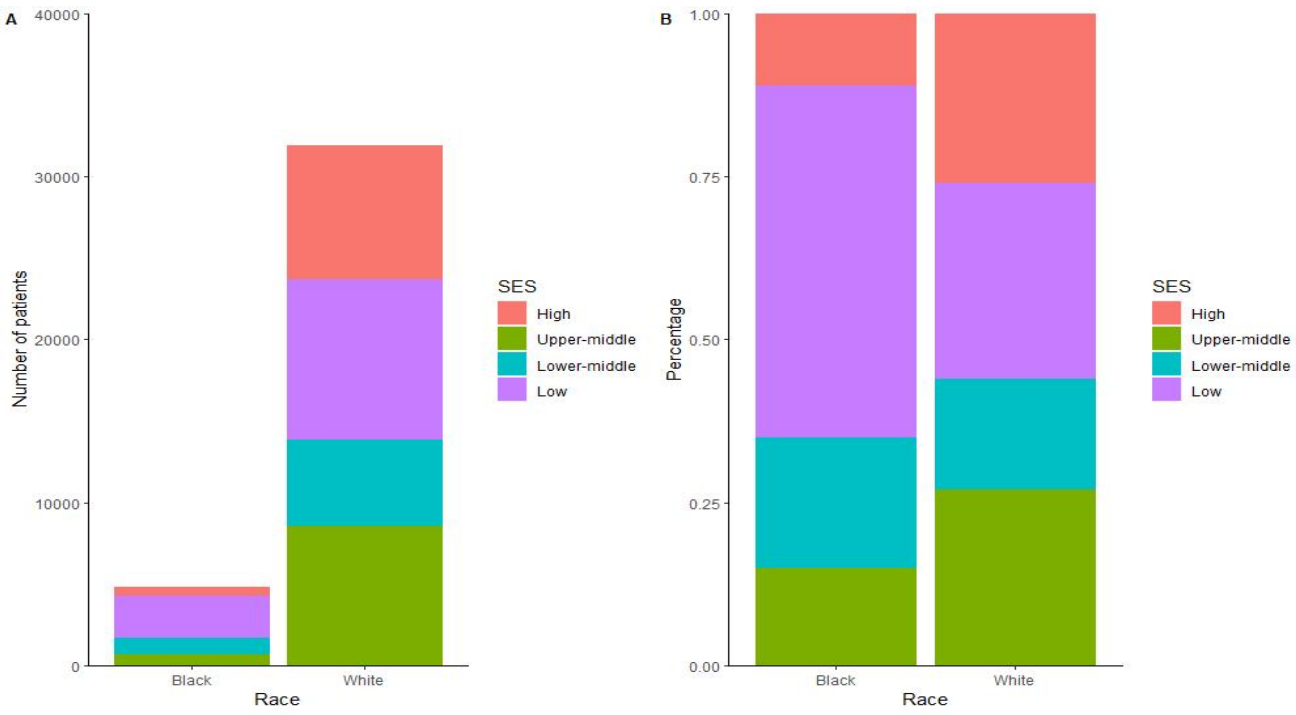

Supplementary Figure 2. Economic status distribution of pancreatic cancer patients of different races from 2002 to 2016.
